# Assessing emergency obstetric care provision in low- and middle-income countries: a systematic review of the application of global guidelines

**DOI:** 10.3402/gha.v9.31880

**Published:** 2016-08-05

**Authors:** Aduragbemi Banke-Thomas, Kikelomo Wright, Olatunji Sonoiki, Oluwasola Banke-Thomas, Babatunde Ajayi, Onaedo Ilozumba, Oluwarotimi Akinola

**Affiliations:** 1 Centre for Reproductive Health Research and Innovation, Lagos State University College of Medicine, Ikeja, Lagos, Nigeria; 2 Department of Community Health and Primary Health Care, Lagos State University College of Medicine, Ikeja, Lagos, Nigeria; 3 Department of Obstetrics and Gynecology, Lagos State University College of Medicine, Ikeja, Lagos, Nigeria

**Keywords:** emergency obstetric care, EmOC assessment, maternal health, quality of care, low- and middle-income countries

## Abstract

**Background:**

Lack of timely and quality emergency obstetric care (EmOC) has contributed significantly to maternal morbidity and mortality, particularly in low- and middle-income countries (LMICs). Since 2009, the global guideline, referred to as the ‘handbook’, has been used to monitor availability, utilization, and quality of EmOC.

**Objective:**

To assess application and explore experiences of researchers in LMICs in assessing EmOC.

**Design:**

Multiple databases of peer-reviewed literature were systematically reviewed on EmOC assessments in LMICs, since 2009. Following set criteria, we included articles, assessed for quality based on a newly developed checklist, and extracted data using a pre-designed extraction tool. We used thematic summaries to condense our findings and mapped patterns that we observed. To analyze experiences and recommendations for improved EmOC assessments, we took a deductive approach for the framework synthesis.

**Results:**

Twenty-seven studies met our inclusion criteria, with 17 judged as high quality. The highest publication frequency was observed in 2015. Most assessments were conducted in Nigeria and Tanzania (four studies each) and Bangladesh and Ghana (three each). Most studies (17) were done at subnational levels with 23 studies using the ‘handbook’ alone, whereas the others combined the ‘handbook’ with other frameworks. Seventeen studies conducted facility-based surveys, whereas others used mixed methods. For different reasons, intrapartum and very early neonatal death rate and proportion of deaths due to indirect causes in EmOC facilities were the least reported indicators. Key emerging themes indicate that data quality for EmOC assessments can be improved, indicators should be refined, a holistic approach is required for EmOC assessments, and assessments should be conducted as routine processes.

**Conclusions:**

There is clear justification to review how EmOC assessments are being conducted. Synergy between researchers, EmOC program managers, and other key stakeholders would be critical for improved assessments, which would contribute to increased accountability and ultimately service provision.

## Introduction

Although maternal mortality has declined by almost 50% since the 1990s, the rate of decline has been slow, as an estimated 800 women still die daily from avoidable pregnancy-related causes. About 99% of these deaths occur in low- and middle-income countries (LMICs) (1, 2), which are also known as developing countries (3). In these countries, maternal mortality remains a major public health challenge with hemorrhage, hypertension, obstructed labor, infection, and complications of unsafe abortion leading to more than three-quarters of maternal deaths (2).

**Fig. 1:**
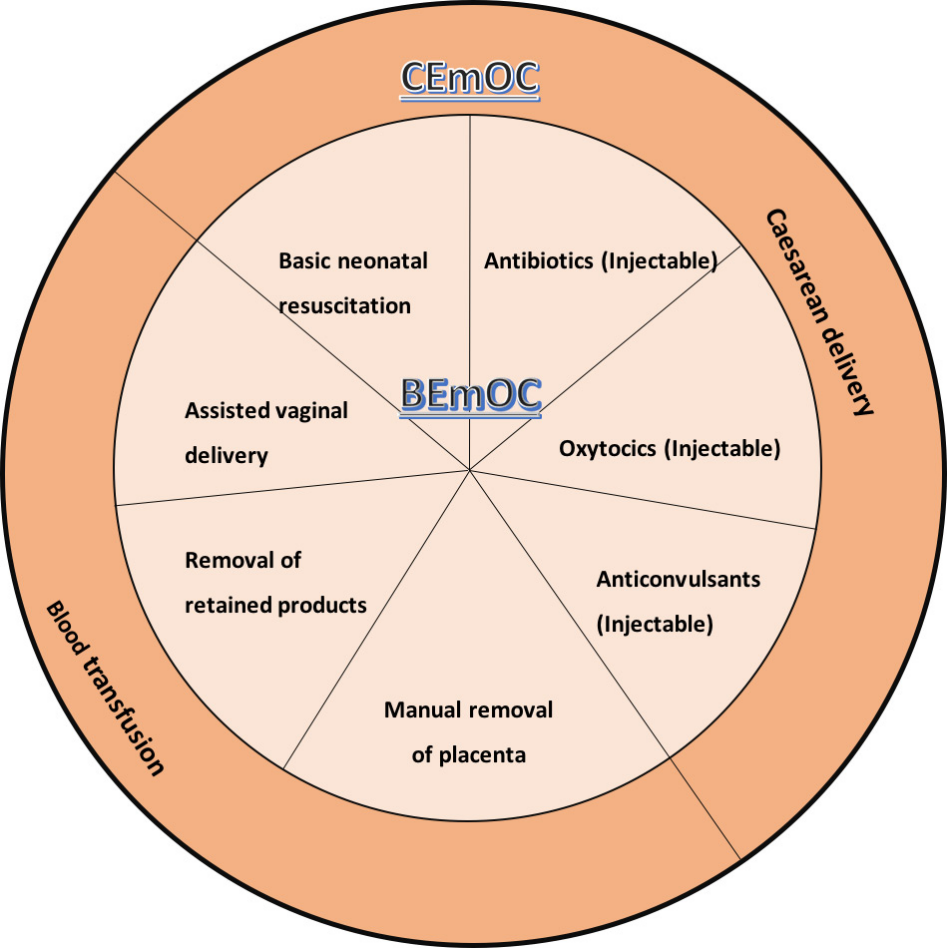
EmOC signal functions.

Evidence suggests that provision of timely and quality emergency obstetric care (EmOC) by a skilled health care professional can potentially reduce the maternal morbidity and mortality that would otherwise occur (4, 5). EmOC refers to ‘care provided in health facilities to treat direct obstetric emergencies that cause the vast majority of maternal deaths during pregnancy, at delivery and during the postpartum period’ (6). To monitor the availability, utilization, and quality of EmOC services, a set of guidelines, first issued in 1992 and finally published in 1997, was developed by experts from the Mailman School of Public Health at Columbia University, with support from the United Nation's Children Funds (UNICEF) and the World Health Organization (WHO) (7). This guideline proposed eight different care packages, referred to as ‘signal functions’, which were described as lifesaving. Six of the eight care packages constituted basic emergency obstetric care (BEmOC): antibiotics (injectable), oxytocics (injectable), anticonvulsants (injectable), manual removal of placenta, removal of retained products, and assisted vaginal delivery. These six care packages in addition to the provision of caesarean and blood transfusion services make up comprehensive emergency obstetric care (CEmOC). In this guideline (7), six indicators, as well as the type of data required to construct these indicators and minimum and/or maximum acceptable standards, were set. Incorporating evidence from the field and literature, the guidelines were reviewed and updated in 2009 (8). In the updated guideline (version 2.0), referred to as a ‘handbook’ by the WHO and partners ‘to emphasize its practical nature’ (8), one more signal function – basic neonatal resuscitation – was added to the BEmOC package, bringing it to a total of seven BEmOC signal functions and nine signal functions in all (8) (Fig. 1). Facilities are classified as BEmOC or CEmOC based on their actual performance of the signal functions in the past 3 months. In this update, although refining some of the previously listed indicators, two new indicators were added, making a new total of eight indicators (Table 1). Similarly, background of the indicators, type of data required, minimum and/or maximum acceptable standards, data collection and analysis, and interpretation and presentation of results were suggested (8).

**Table 1:** EmOC indicators with acceptable levels

	Indicator	Acceptable level
1.	Availability of emergency obstetric care: basic and comprehensive care facilities	There are at least five emergency obstetric care facilities (including at least one comprehensive facility) for every 500,000 population.
2.	Geographical distribution of emergency obstetric care facilities	All subnational areas have at least five emergency obstetric care facilities (including at least one comprehensive facility) for every 500,000 population.
3.	Proportion of all births in emergency obstetric care facilities	Minimum acceptable level to be set locally.
4.	Met need for emergency obstetric care: proportion of women with major direct obstetric complications who are treated in such facilities	100% of women estimated to have major direct obstetric complications are treated in emergency obstetric care facilities.
5.	Caesarean sections as a proportion of all births	The estimated proportion of births by caesarean section in the population is not less than 5% or more than 15%.
6.	Direct obstetric case fatality rate	The case fatality rate among women with direct obstetric complications in emergency obstetric care facilities is less than 1%.
7.	Intra-partum and very early neonatal death rate^a^	Standards to be determined.
8.	Proportion of maternal deaths due to indirect causes in emergency obstetric care facilities^a^	No standard can be set.

a New indicators added in the updated handbook.

The ‘handbook’ has been used by many program planners and managers for many needs assessments, both at district and national levels (8). A toolkit consisting of 10 modules was also developed by the Averting Maternal Death and Disability (AMDD) program of Columbia University to support planning and conduct of these needs assessments (9). As of 2011, more than 70 needs assessments had been completed at subnational and national levels (10). The ‘handbook’ has also been used by researchers to elucidate the sufficiency or otherwise of EmOC in several countries. However, to the best of our knowledge, there has not been any systematic review of the literature that captures the application of this handbook and/or experiences of researchers in applying the handbook in assessing EmOC. We believe that the importance of such review lies in its potential to extricate lessons learnt and best practices that have been effective while unraveling key gaps that need to be addressed in framing a revised ‘handbook 3.0’ going forward. Our objective in this review was to explore and critically appraise the use of the handbook 2.0 (8) while capturing the experiences of researchers in assessing EmOC in LMICs.

## Methods

We used the Preferred Reporting Items for Systematic Reviews and Meta-Analyses (PRISMA) approach (11) to report findings of this systematic review of studies assessing EmOC performance in LMICs (see Supplementary File 1).

### Search strategy

We conducted a preliminary search on Google Scholar to test the sensitivity of the proposed search terms and to explore other possible search terms that could also be used to identify relevant studies for inclusion in our review. Thereafter, we searched Scopus, PubMed, CINAHL, PsycINFO, Embase, Global Health, and Directory of Open Access Journal (DOAJ) for articles published after 2008 (to capture 1 year before the updated handbook (8) was published) till end of June 2016 (when we closed the search), using the following search terms:

“Emergency Obstetric Care” OR “Emergency Obstetric and Newborn Care” OR EmOC OR EmONC.

AND

Assess* OR describe* OR monitor* OR evaluate* OR function* OR perform* OR effect* OR impact OR outcome.

(We used both EmOC and EmONC for completeness because both terminologies are commonly used interchangeably (8)).

We identified and removed duplicates from the results retrieved from all databases. We complemented the results of our search with reference-list checking of the articles that we retrieved. We did this in order to identify any additional relevant articles that may have been missed during the automated search.

Three co-authors (ABT, KW, and OS) independently conducted the search. All three authors reviewed all records that were retrieved and subsequently agreed on the final eligibility of the retrieved articles based on established inclusion and exclusion criteria. Any disagreements were resolved by the fourth co-author (OI).

### Inclusion and exclusion criteria

Articles were included if they reported observational studies that described or assessed the provision of EmOC service and were retrieved from peer-reviewed sources. Only studies that were published in English or French language were included in this systematic review. In addition, the study must have been conducted in an LMIC, as classified by the World Bank (3).

Articles that were editorial letters, commentaries, or non-systematic reviews were excluded from our review.

### Data extraction and synthesis

Following retrieval, all included papers were allocated unique identifiers for audit purposes. The full texts of the included papers were reviewed and data were collected in a pre-developed extraction sheet.

The pre-developed data extraction tool was used to extract data on the author(s), publication year, country in which the study was conducted, study design, scale of the study (national, subnational, or facility level), specific study site(s), number of facilities studied, statement of study objective(s), data source(s) used, collection of EmOC indicators, process of data collection for EmOC indicator(s), methodological limitations captured, and recommendations made to improve future EmOC assessments.

We used thematic summaries (12, 13) to summarize our findings from the included studies. We subsequently mapped patterns that we observed in the assessment or description of EmOC service provision in LMICs. To analyze methodological limitations and lessons learnt from conducting EmOC assessments, we took the deductive approach of the framework synthesis (12, 13) presenting our findings as emerging key themes.

### Quality assessment

As there was no previously existent quality assessment checklist, we developed a 23-criteria checklist across the eight EmOC indicators (Table 2), leveraging best practices suggested in the ‘handbook’ (8).

**Table 2:** Quality assessment checklist for EmOC assessment

Quality criteria for indicators
Indicator 1: Availability of EmOC
Compared (total or representative) number of functioning facilities with the most recent population size (or projected population if recent population size is older than 5 years).
Included all facilities within the relevant geographical level (national, district, subdistrict): Public and private.
Direct inspection to collect data.
Indicator 2: Geographical distribution of EmOC facilities
Geo-referenced EmOC facilities and identified catchment population for the facility.
Identified underserved areas using disaggregated data.
Included public and private.
Indicator 3: Proportion of all births in EmOC facilities
Triangulated with parallel indicator – proportion of institutional deliveries.
Used most recent population size (or projected population if recent population size is older than 5 years).
Used disaggregated data to relevant geographical level (national, district, subdistrict).
Indicator 4: Met need for EmOC
Triangulated with parallel indicator – proportion of institutional deliveries.
Adhered to operational definition of direct obstetric complications.
Defined period for which data on women treated for direct obstetric complications were collected.
Used most recent population size (or projected population if recent population size is older than 5 years).
Used disaggregated data to relevant geographical level (national, district, subdistrict).
Indicator 5: Caesarean sections as a proportion of all births
Triangulated with parallel indicator – proportion of institutional deliveries.
Used denominator as expected number of live births (in the whole catchment area, not just in institutions).
Used disaggregated data to relevant geographical level (national, district, subdistrict).
Indicator 6: Direct obstetric case fatality rate
Triangulated with parallel indicator – proportion of institutional deliveries.
Used as numerator data of women who developed direct obstetric complications after admission, and die before discharge.
Used as denominator number of women who were treated in the same facility and over the same period as numerator.
Calculated cause-specific fatality rates for each of the major causes of maternal death.
Indicator 7: Intrapartum and very early neonatal death rate
Used fresh stillbirths (intrapartum and very early neonatal deaths within the first 24 h) as numerator.
Denominator used was all women who gave birth in the facility during the same period.
Newborns under 2.5 kg were excluded from the numerator and the denominator.
Indicator 8: Proportion of deaths due to indirect causes in EmOC facilities
Used data on ‘previous existing disease or disease that developed during pregnancy and which was not due to direct obstetric causes, but which was aggravated by the physiologic effects of pregnancy.
Used as denominator all maternal deaths in the same facilities during the same period.
Used disaggregated data to relevant geographical level (national, district, subdistrict).

One point was recorded for each criterion observed to have been ‘achieved’ and 0 points were recorded if the item was ‘not achieved’. If it was unclear whether the specific criterion had been achieved or not, ‘CT’ (‘could not tell’) was recorded. For articles that did not report a particular indicator as part of their objectives in the first place, it was recorded as ‘NA’ (‘not applicable’).

Articles were classified as high quality, if they achieved 75% or more of the criteria relevant for the specific indicator(s) that the authors reported in their study. Medium quality articles achieved between 50 and 74%, whereas low quality articles were those which achieved less than 50%.

## Results

### Summary of results

**Fig. 2:**
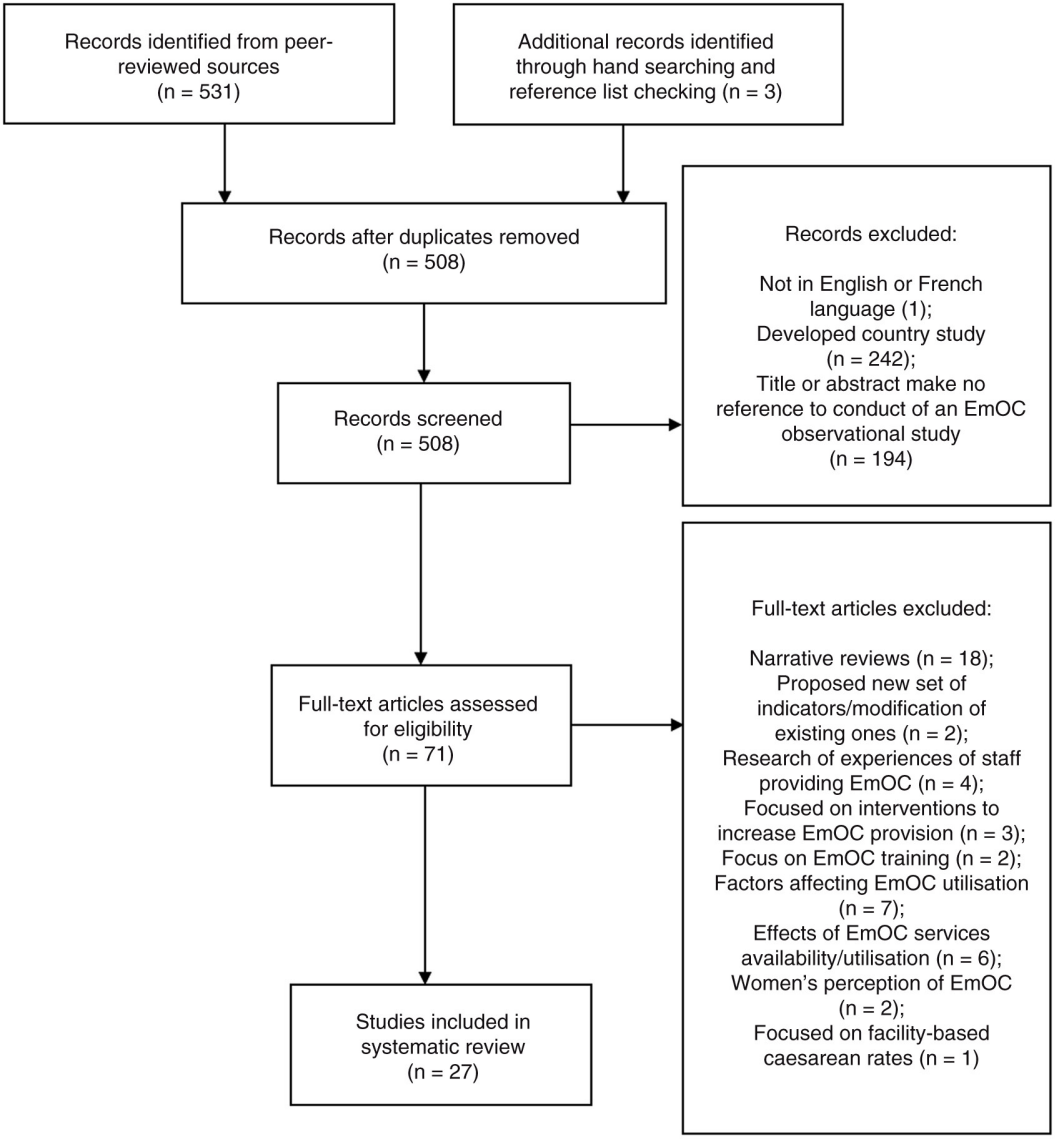
PRISMA diagram showing search process.

We retrieved 508 records after removal of duplicates. Following the abstract and full-text reading, 27 studies which met the inclusion criteria were included for review (Fig. 2).

### Quality assessment of the included studies

Eighteen of the 27 studies were adjudged to be of high quality (14–31), five were of medium quality (32–36), and another four were adjudged to be of low quality (37–40) (see Supplementary File 2).

### Distribution of EmOC assessments published in peer-reviewed literature

**Fig. 3:**
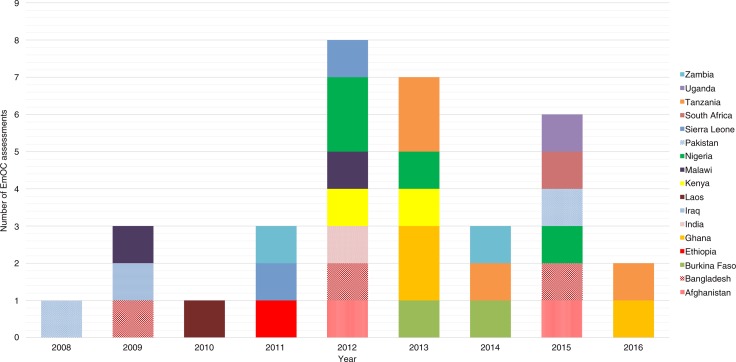
Distribution of EmOC assessments conducted since 2009.

Following the launch of the handbook in 2009, assessments of EmOC provision steadily increased, peaking in 2012. Following a noticeable decline in evaluations in 2014, there was an immediate increase in 2015 (Fig. 3). An average of four EmOC assessments were conducted annually, which were published in peer-reviewed literature.

Of the EmOC assessments included in our study, four were conducted in Nigeria (14, 17, 28, 30) and Tanzania (22, 24, 38, 40), three each have been conducted in Bangladesh (16, 17, 33) and Ghana (18, 34, 38), and two each in Afghanistan (25, 26), Burkina-Faso (19, 38), Kenya (17, 21), Malawi (17, 27), Pakistan (32, 36), Sierra Leone (17, 22), and Zambia (23, 29). One assessment was conducted each in Ethiopia (15), India (17), Iraq (37), Laos (20), South-Africa (39), and Uganda (31) (Fig. 3).

### Characteristics of EmOC assessment studies in LMICs

One study was published in 2008 (32) and three in 2009 (27, 33, 37). Since 2012, there have been a minimum of three studies per year, with three studies published in 2012 (17, 26, 28), and five in 2013 (21, 24, 30, 34, 38). The highest number of studies for a year (six) was published in 2015 (14, 16, 25, 31, 36, 39). By the close of the search, two studies had been published in 2016 (18, 22).

Seven studies were conducted across all facilities at a national level (15, 16, 18, 19, 23, 26, 35); 17 studies were conducted at a subnational level, within a district or a collection of many facilities (14, 17, 20–22, 24, 27, 29–34, 36–39), while three studies were conducted within a facility (25, 28, 40) (Table 3). The total number of facilities assessed by authors in the various studies ranged from 1 (25) to 2,387 (16) (see Supplemental File 3).

**Table 3:** Summary of study characteristics

Study characteristics	No. of studies (*n*=27)	% of total
Scale of study		
National	7	25.9
Subnational	17	63.0
Facility	3	11.1
Assessment model		
UN EmOC assessment tool	23	85.2
UN EmOC assessment tool + another tool	2	7.4
Geographic information system framework	1	3.7
Quality of care assessment tool	1	3.7
Study design		
Cross-sectional facility-based survey	17	63.0
Mixed methods (facility data + interviews with healthcare provider)	8	29.6
Mixed methods (secondary data + primary geographical data collection)	1	3.7
Mixed methods (interviews and primary geographical data collection)	1	3.7
Indicators collected		
Indicator 1: Availability of EmOC services		
Fully collected	20	74.1
Partially collected (signal functions only)	6	22.2
Not collected	1	3.7
Indicator 2: Geographical distribution of EmOC facilities		
Collected	9	33.3
Not collected	18	66.7
Indicator 3: Proportion of all births in EmOC facilities		
Collected	11	40.7
Not collected	16	59.3
Indicator 4: Met need for EmOC		
Collected	10	37.0
Not collected	17	63.0
Indicator 5: Caesarean sections as a proportion of all births		
Collected	14	51.9
Not collected	13	48.1
Indicator 6: Direct obstetric case fatality rate		
Collected	11	40.7
Not collected	16	59.3
Indicator 7: Intrapartum and very early neonatal death rate		
Collected	3	11.1
Not collected	23	85.2
Indicator 8: Proportion of deaths due to indirect causes in EmOC facilities		
Collected	3	11.1
Not collected	22	81.5

Twenty-three studies used the WHO EmOC assessment tool alone (14–23, 26–37, 39, 40). Two studies combined the WHO EmOC assessment tool with some other quality assessment tool. One of these studies (38) used a tool that focused on interpersonal and technical performance and continuity of care (41) and satisfaction of patients (42), whereas the other study (24) incorporated the Safe Motherhood Needs Assessment framework. One other study (34) used a quality of care assessment tool that captured non-medical quality indices and another one used only geographical indices within a geographic information system (GIS) framework (25) (Table 3).

Seventeen studies collected data for EmOC assessment by conducting cross-sectional facility-based surveys (14, 15, 17, 18, 21, 24, 26–28, 30–32, 34–36, 39, 40). Eight studies used mixed methods, collecting facility data and conducting interviews with health care providers (16, 19, 20, 22, 29, 33, 37, 38). Another study also used mixed methods, but combined secondary facility data with primary geographical data collection (23). The final study included in our review used a combination of interviews with primary geographical data collection (25).

In terms of indicators captured, 20 studies reported Indicator 1 fully, including availability of EmOC facilities and signal functions (14–24, 27, 28, 31–33, 35–37). Six studies captured Indicator 1 partially, by reporting availability of signal functions alone (26, 29, 34, 38–40). One study did not report on Indicator 1 at all (25) (Table 3). Nine studies captured geographical distribution of EmOC facilities (Indicator 2) (15, 18, 19, 21–23, 25, 27, 35). Eleven studies reported proportion of all births in EmOC facilities (Indicator 3) (14, 15, 17, 20, 22, 26–28, 30, 32, 35). Ten studies reported met need for EmOC (Indicator 4) (14, 15, 17, 20, 22, 26, 27, 31, 35, 36). Caesarean sections as a proportion of all births (Indicator 5) was reported in 14 studies (14, 15, 17, 19, 20, 22, 26, 27, 30–33, 35, 36), while 11 studies reported direct obstetric case fatality rate (Indicator 6) (14, 15, 17, 20, 22, 26, 27, 30, 31, 35, 36). Three studies each reported intrapartum and very early neonatal death rate (Indicator 7) (14, 22, 36) and proportion of deaths due to indirect causes in EmOC facilities (Indicator 8) (14, 15, 26) (Table 3).

### Experiences of researchers in EmOC assessments and recommendations for future improvements

Four key themes emerged from our analysis, namely: improving data quality for EmOC assessments, refining indicators for subsequent guidelines for EmOC assessments, holistic approach required for EmOC assessments, and integrating the EmOC assessments as part of routine process.

#### Improving data quality for EmOC assessments

Issues such as lack of/incomplete database on obstetric emergencies (35) and inaccuracies of data such as early neonatal deaths being recorded as stillbirths were reported by multiple authors (15, 30).

Data quality was poor and no data on obstetric complications were recorded …. (35)

Incompleteness of death records led to 10% of causes of death remaining unknown, which may have caused under or overestimation of some causes. Furthermore, early neonatal deaths are sometimes recorded as stillbirths and it is evident that a few regions seriously underreported maternal deaths and both stillbirths and early neonatal deaths. (15)

One study conducted across multiple districts in six developing countries, attributed these issues to poor record keeping in facilities, particularly as it relates to the complications for which women present (17). This poor record keeping affects results of indicator estimates (17, 20) and ultimately limits the quality of EmOC assessments that are conducted with such data.

Data on the number of women with EmOC complications are not currently routinely collected in most labor ward registry books – although the number of deliveries and number of CS (caesarean sections) are generally accurately recorded. This will affect estimates provided of the met need for EmOC as well as case fatality rates. (17)

To address the issue of data quality at the health system level, Ameh et al., who conducted their assessment through focus group discussions with health care providers, recognized that non-triangulation of their findings was a limitation of their research (37). It is well established that triangulation of multiple sources of data helps to improve data quality, as well as confidence, accuracy, and reliability in results (43). In our review, some authors triangulated data from facility registers with direct observation of the equipment and drugs available for each signal function (21). Others combined quantitative and qualitative data (16, 19, 20, 29, 33, 37, 38).

A review of facility registers to ascertain that the signal functions were performed was done. In addition, observations to indicate the availability of equipment and drugs (for each signal function) were conducted. (21)

Another study suggested that incorporating process indicators and leveraging computer systems for data entry would help improve data quality for EmOC assessments (37).

More must be done to integrate the UN Process Indicators from the start of projects to monitor and evaluate EmOC services … improved their data collection systems by upgrading to computers. (37)

In addition, training of data collectors prior to the start of their survey (27) and using local language (24) to conduct the survey were identified as some other best practices that could be explored to improve the data quality of EmOC assessments.

#### Refining indicators for subsequent guidelines for EmOC assessments

Some authors in our review suggested the need to refine the current EmOC indicators in the ‘handbook’ (8) when preparing future updates. This is to allow future assessments to give more relevant information for decision-making.

Regarding availability of EmOC (Indicator 1), in addition to the recommended number of facilities per 500,000 population, Douangphachanh et al. reported on population density (20). To refine Indicator 1, Bosomprah et al. suggested that number of EmOC facilities per number of births and/or estimated number of pregnancies in the population are a better reflection of the EmOC requirements of the population (18). Some other authors reported on the availability of human resources for EmOC services (24, 28, 34). For example, Nesbitt et al. (34) reported on the number of health workers available to perform the various signal functions, whereas some other authors reported on availability of health workers for 24-h EmOC provision, which suggest that this added invaluable information could be crucial while assessing the true availability of EmOC services (23, 28).

… births and estimated pregnancies are a better metric of need than population. This perspective shows that the prioritized interventions if implemented, could increase the number of EmONC facilities dramatically. (18)

… assessing health worker density including 24/7 availability, electricity and geographic access adds crucial information. (23)

For Indicator 2, factors such as travel condition of roads and rivers, including consideration for traffic and travel times as well as altitude and elevation of the area were suggested as critical information that should be captured (23, 25).

The geographic analyzes could be refined by using population figures for areas smaller than wards and considering geographic data on roads, rivers, and altitude. Simultaneously assessing health worker density including 24/7 availability, electricity, and geographic access adds crucial information. (23)

It may have been necessary to consider traffic flows and congestion in modeling urban travel times. (25)

Admasu et al. suggested that GIS data should be considered as providing critical evidence that will be useful in deciding the optimum location of EmOC facilities and in developing robust referral systems (15), especially as pregnant women have to travel to these facilities.

It is recommended that: geocoded spatial analyses are used to rationalize decisions regarding location of new or upgraded facilities and to develop referral systems. (15)

Echoka et al. estimated the mean distance that women had to travel to reach CEmOC facilities (21).

#### Holistic approach required for EmOC assessments

Bearing in mind the multifaceted aspects of care that mothers and their children require during delivery, some authors have proposed a holistic approach for future EmOC assessments (34). It has been suggested that this approach should capture both medical needs (technical know-how for patient care) and non-medical needs (responsiveness of care (44) received by patients, that is, mother and child).

There are several dichotomous elements to consider in maternity care that complicate the operationalization of quality assessments: two recipients (mother and child), two aspects of care (medical and non-medical) and two modes of care (routine and emergency). We advocate that quality assessments of maternal and newborn care acknowledge these and adopt a holistic approach. (34)

#### Integrating the EmOC assessments as part of routine process

In another study, Ameh et al. suggested that EmOC assessments should be conducted as a routine process and not just as a component of project monitoring and evaluations (17). These routine assessments should be done bearing in mind the potential for the Hawthorne effect, which may positively affect health care provider behavior though the presence of an observer is deemed to be short-lived to between 10 and 15 observations (38).

Pairs of interviewers visited every facility without prior notice. Revisits were not undertaken if the facility was closed. (24)

The other mode of assessment is to use existing databases. For example, Bosomprah et al. used a nationwide cross-sectional facility-based survey that included both public and private facilities that recorded at least five deliveries per month in 2009 using data from an existing district health management information system (18).

## Discussion

This systematic review has helped to map EmOC assessments conducted in LMICs which have been published in peer-reviewed journals since 2008, about the time the updated handbook (8) was released. This review has also described the scale of the EmOC assessments conducted, type of assessment frameworks used, type of data collected, as well as indicators captured. In addition, we synthesized information regarding experiences of researchers and recommendations proffered by authors for future EmOC assessments based on their field experience.

### Limitations

This review needs to be interpreted bearing in mind the following limitations. Firstly, we have only included EmOC assessments that were published in peer-reviewed literature. It is highly likely that there are some unpublished EmOC assessments that exist as national or subnational reports, which may or may not be available in the public domain. Although we recognize that this may be a limitation, we were constrained by the enormity of the task of having to reach out to all the relevant bodies (international, national, and local) to request for any EmOC assessments that they may have conducted. However, we do not believe that the interpretations given to our findings or the conclusions made would have been altered otherwise, because the same EmOC assessment framework would have been used in assessing EmOC provision in those reports.

Secondly, we could not retrieve any previously designed quality checklist for assessing study quality. As such we designed a 23-criteria checklist (Table 2). Although we recognize the possible weaknesses in our proposed quality assessment framework, we opine that by developing the quality checklist based on recommendations proposed by the WHO (8), it reflects the insights of the global community of experts that prepared the ‘handbook’ in the first place. Thus, we believe that it provides a basis for more formalized development of subsequent quality assessment and accountability frameworks for EmOC assessment studies.

### Quality of EmOC assessment studies in LMICs

Unlike the studies done at subnational scale, all the studies done on a national scale were adjudged as being of high quality. The underlying reason for this was not particularly clear. However, we believe that this is plausible because such studies were conducted using large databases that afforded the researchers the ability to capture all required data in answering their research questions. In the post-2015 era, emphasis is being placed on the need to capture disaggregated data that would allow for identifying areas of most need, type of need in those areas, and how best to implement interventions that address those needs (45). As such, there is the need for more ‘high quality’ EmOC assessments at subnational levels. This will inherently lead to the generation of robust subnational level datasets that can provide meaningful and helpful information to guide policymakers and program managers to better plan EmOC service provision.

Specifically, Indicator 1 (availability of EmOC) and Indicator 7 (intrapartum and very early neonatal death rate) were the two indicators that lowered quality scores the most. For Indicator 1, the major problem with studies assessed as being of low quality was the non-comparison of total or representative number of functioning facilities with the most recent population size (or projected population if recent population size is older than 5 years) and the non-inclusion of all facilities within the relevant geographical level (national, district, subdistrict), including public and private hospitals. For Indicator 7, the main issues were non-capture of fresh stillbirths alone and non-exclusion of newborns under 2.5 kg, as recommended in the ‘handbook’ (8).

### Conduct of EmOC assessments in LMICs

Our findings showed that since 2008, there has generally been steady interest in EmOC assessments, mostly because of donor-funded projects and programs. In more recent times, 2015 marked the highest number of publications of EmOC assessments in peer-reviewed literature. Although, the reason for this increased interest is not particularly clear, through further investigation, we observed that half of the assessments were part of a large Department for International Development (DFID) funded EmOC training program, which had an EmOC assessment component, from which articles were then published for knowledge sharing purposes (46).

Our findings revealed that the ‘handbook’ has been the most widely used guide for EmOC assessments. However, some authors have tried to capture other components of the care that they deemed important. Quality metrics such as satisfaction of patients (42), interpersonal (provider attitude) and technical (provider skill) performance, continuity of care (41), and broader geographical indices (25) were incorporated in a few studies. Going forward, we believe that combining some of these metrics with the existing indicators from the ‘handbook’ during EmOC assessment can provide credible insights into gaps in the present framework that need to be bridged. An adoption of this ‘holistic’ approach is deemed timely and appropriate especially in aligning with EmOC assessments’ need for the post-2015 era, where there is a resounding interest in subjective well-being (45).

Two-thirds of the included studies conducted a cross-sectional facility-based survey to collect data for EmOC assessments. However, expanding both at the point of assessment by using mixed methods and expanding linearly by monitoring trends will improve the value of EmOC assessments. As seen in seven studies that adopted a mixed method approach (16, 19, 20, 29, 33, 37, 38), collecting facility data and conducting interviews with health care providers for EmOC assessments allows researchers to capture broader issues regarding EmOC service provision. Linear assessments, where EmOC service provision at different time periods are compared, allow detection of trends in the capacity of hospitals to provide the signal functions (19). On the other hand, qualitative enquiries such as in-depth interviews and focus groups would be useful in understanding the ‘why?’ For example, ‘why particular signal functions are not performed’ (22).

### The EmOC indicators

Availability of EmOC facilities (Indicator 1) is the most widely reported of all the EmOC indicators. Full reporting of Indicator 1 requires capturing both the number of facilities per 500,000 population and the availability of the various signal functions. Although 17 studies reported on the indicator fully, seven studies only reported the signal functions. Not estimating the number of EmOC facilities available per 500,000 population is comprehensible if the sample of facilities selected did not include all the facilities available for the population (38) or in a situation where only a handful of facilities were selected for the assessment in the first place (40). However, it is not clear why some of the studies (29, 34, 39) have not estimated the ratio because these studies had captured all facilities within a defined population area.

There are two challenges with Indicator 1, highlighted by authors in our review. Firstly, there is the challenge of populations less than 500,000 (28). Kongnyuy et al. utilized the number of facilities per 125,000 population, because there were some populations in their chosen defined geographical area which were less than 500,000 (27). Secondly, although the 500,000 population provides a sufficient basis for comparison of EmOC availability, it does not reflect the actual need for the population. Bosomprah et al. suggested that the number of EmOC facilities per number of births and/or the estimated number of pregnancies in the population are a better reflection of the EmOC requirements of the population (18), as opposed to the 500,000 population denominator. The ‘handbook’ explained that the reason why the minimum acceptable level for Indicator 1 was defined in relation to the population size rather than number of births is because ‘most health planning is based on population size’. It, however, goes on to suggest that ‘If it is judged more appropriate to assess the adequacy of EmOC services in relation to births, the comparable minimum acceptable level would be five facilities for every 20,000 annual births’ (8). This benchmark needs to be equally highlighted, pointing out its capacity to reflect ‘actual need’ (18). Furthermore, our review showed that some confounding factors of availability such as population density (20), availability of human resources for EmOC services (24, 28, 34), and 24 hours a day/7 days a week service provision (23, 28) need to also be considered in reporting this indicator.

Regarding the performance of signal functions themselves, a majority of the authors in our review reported this differently. Although some reported signal function performance in 3 months (27), others reported conduct within a 6-month period (24). Echoka et al. suggested that the recommended 3-month assessment period be extended to a 6-month period in districts with low facility deliveries (21). Other authors recognized that the actual performance of signal functions bordered on several factors. Mezie-Okoye et al. concluded that signal functions that required ‘supply of medical consumables were performed by more facilities than those that required special training, equipment, and maintenance’ (28). We surmise that going forward, there is a need to capture signal function performance based on three indices critical for its conduct: drugs, equipment, and personnel. This is especially important considering that issues bordering on health systems failures in providing equipment for care and inadequate human resources have been previously reported as key contributors to the gaps in the provision of EmOC (47).

Based on findings from our review, nine studies reported Indicator 2. When the ‘handbook’ was initially published, there was anticipation that digital mapping and GIS would become more widely available (8). Indeed, there has since been a global expansion in GIS capabilities and application, even in developing countries in Africa and Asia (48). GIS allows us to ‘visualize, question, analyze, and interpret data to understand relationships, patterns, and trends’ (49). In our review, we observed that in using GIS, researchers have estimated straight line distances (‘as the crow flies’) between facilities and place of abode of women (25) and built buffer zones around facilities to reflect coverage (23), both of which do not reflect the real-life travel experiences of women. Clearly, there is a need for greater leverage on the potential benefits that GIS offers (15). However, we recognize that the low reporting of this indicator may be because it requires specialized knowledge and skill on the use of GIS software such as ESRI ArcGIS, MapInfo, GRASS GIS, QGIS, etc. EmOC assessors should consider collaborations with GIS experts and urban planners in integrating GIS research components in future assessments. Some authors have suggested that going forward factors such as travel condition of roads and rivers, including consideration for traffic and travel times as well as altitude and elevation of the area have to be captured to provide more informative evidence that can aid decision makers (25, 23). Similar suggestions were proffered by authors that explored barriers to formal EmOC utilization (50). The point on population density made in one of the studies (20), had been highlighted in the handbook (8), in which a suggestion was made that ‘where the population is widely dispersed… it may be advisable for governments to exceed the minimum acceptable’. If so, by how much? In a time of limited public resources, where focus is on demonstrating value for money (51), exceeding the minimum acceptable would mean upgrading the existing facilities and building new facilities in the most optimum sites to improve outcomes for mothers and their babies.

For the remaining indicators (Indicator 3–8), these essentially rely on robust data systems. To implement such data systems, it is critical to incorporate process indicators in routine monitoring processes, while leveraging computer systems for data entry which would help improve data quality for EmOC assessments (37). Of these indicators (3–8), the least reported two were intrapartum and very early neonatal death rate (Indicator 7), which was reported in two studies (14, 36) and proportion of deaths due to indirect causes in EmOC facilities (Indicator 8), which was reported in three studies (14, 15, 26). These two indicators are also the same for which standards have not been determined (8). The main issue identified with Indicator 7 is its requirement to differentiate fresh from macerated stillbirths. This may be one of the reasons why several hospital records in LMICs do not report this indicator, as the facilities rarely capture this differentiation in a systematic fashion (15). Secondly, the definition of very early neonatal death ‘a death that occurs within the first 24 hours of life’ (8) may be challenging in itself given that the majority of mothers would usually have been discharged by 6 to 12 h after delivery (15, 52). As such, studies that assessed this indicator reported the number of stillbirths alone as a surrogate for Indicator 7 (17, 36) or reported stillbirths in addition to deaths that occurred before the mother's discharge (15). For Indicator 8 there was no clear indication regarding its low reporting, which may be because of the poor data systems reported in many developing countries and the difficulty in identifying indirect deaths, which leads to underestimation of the numbers (15, 26).

### Going forward

To improve data quality for EmOC assessments, we opine that multiple strategies are required. As some of the authors in our review have suggested, there is a need to integrate EmOC assessments as part of the routine process of monitoring and evaluation (17), and not just when programs are being conducted. The implementation of the sustainable development goals (SDGs) in the post-2015 era, which is anticipated to be a highly data-intensive period, offers a renewed opportunity to leverage robust, routinely collected, quality data (53). There is a need to incorporate EmOC assessments in the SDG framework including accountability at all levels. Secondly, there have been suggestions for using computerized systems to capture data required for assessments which may help to improve data quality (37). Nonetheless, we believe that a computer without the appropriate personnel to input the data may still be prone to errors. A learning point from developed countries may be the use of appropriately trained perinatal nurses (54), who understand the nuances of EmOC to capture the relevant data for assessments. The ‘handbook’ advised that the data to be used in developing the indicators should either be ‘already available or relatively easy and economical to obtain’ (8).

In addition to training data collectors and implementing a multilayered plan for quality in order to achieve success with EmOC assessments, it is critical to bring together all key stakeholders. Ethiopia's assessment was largely successful because of effective local leadership and a vibrant collaborative process that involved the Ministry of Health, relevant international organizations, representatives from the Ethiopian Society of Obstetricians and Gynecologists, and Columbia University's Averting Maternal Death and Disability Program, who provided ample technical support (55).

## Conclusion

This is not the first attempt to contribute towards efforts to improve future EmOC assessments. Gabrysch et al. proposed a new set of 23 signal functions which incorporate EmONC with routine intrapartum and postnatal care (56). However, we believe that capturing experiences of researchers in assessing EmOC since the last iteration of the global guidance, such as was done by the Maternal Health Task Force and the Global Alliance to Prevent Prematurity and Stillbirth (57) and as has been systematically reported in this review, offers unique insight into how best to proceed with version 3.0 of the handbook.

In the post-2015 era, the SDGs form the basis for development initiatives. The SDG 3 which aims to ‘ensure healthy lives and promote well-being for all at all ages’ has as one of its targets to ‘reduce the global maternal mortality ratio to less than 70 per 100,000 live births’ by 2030 (58). EmOC is central to any strategy aimed at reducing maternal mortality (5). As such, improving frameworks for EmOC assessments will be essential for the new era. Synergy between researchers, EmOC program managers, and other key stakeholders will be critical for these improved assessments. This will contribute to increased accountability and ultimately service provision while driving us closer to reaching the 70 per 100,000 live births target.

## Supplementary Material

Assessing emergency obstetric care provision in low- and middle-income countries: a systematic review of the application of global guidelines

Assessing emergency obstetric care provision in low- and middle-income countries: a systematic review of the application of global guidelines

Assessing emergency obstetric care provision in low- and middle-income countries: a systematic review of the application of global guidelines

## Paper context

In 2009, the World Health Organization updated the guideline for assessing emergency obstetric care provision. However, since its launch, there has been no systematic capture, reported in the literature, of its application and researchers’ experiences using it. Our review offers a systematic analysis of researchers’ experiences in utilizing the guideline in low- and middle-income countries. Findings could potentially be relevant for future assessments and in developing the next global ‘guideline 3.0’.
