# Supplementary material for: Assessing emergency obstetric care provision in low- and middle-income countries: a systematic review of the application of global guidelines
Source: Glob Health Action. 2016 Aug 5;9:10.3402/gha.v9.31880. doi: 10.3402/gha.v9.31880 (PMC4976306; doi:10.3402/gha.v9.31880)
Supplement: Assessing emergency obstetric care provision in low- and middle-income countries: a systematic review of the application of global guidelines [file GHA-9-31880-s002.pdf]

Aduragbemi Banke-Thomas, Kikelomo Wright, Olatunji Sonoiki, Oluwasola Banke-Thomas, Babatunde Ajayi, Onaedo Ilozumba and Oluwarotimi Akinola

Quality criteria for indicators	Abegunde et al. 2015	Admasu et al. 2011	Alam et al. 2015	Ali et al. 2008	Ameh et al. 2009	Ameh et al. 2012	Anwar et al. 2009	Bosomprah et al. 2016	Compaore e al. 2014	Douangphachanh et al. 2010	Duysburgh et al. 2013	Echoka et al. 2013	Fakih et al. 2016	Gabrysch et al. 2011	Hanson et al. 2013	Hirose et al. 2015	Kim et al. 2011	Kongnyuy et al. 2009	Mezie-Okoye et al. 2012	Nesbitt et al. 2013	Owens et al. 2015	Oyerinde et al. 2011	Pattinson et al. 2015	Saidu et al. 2013	Ueno et al. 2015	Utz et al. 2015	Wilunda et al. 2015
**Indicator 1: Availability of EmOC**																											
Compared (total or representative) number of functioning facilities with the most recent population size (or projected population if recent population size is older than 5 years)	1	1	1	1	0	1	1	1	1	1	0	1	1	1	1	NA	0	1	1	0	1	CT	0	1	0	1	1
Included all facilities within the relevant geographical level (national, district, sub-district): Public and private	0	1	1	0	0	1	0	1	1	1	0	1	1	1	1	NA	1	1	1	1	1	1	0	1	1	0	1
Direct inspection to collect data	1	1	1	0	0	1	1	0	1	1	1	1	1	1	1	NA	1	1	1	1	1	1	1	1	0	1	0
**Indicator 2: Geographical distribution of EmOC facilities**																											
Geo-referenced EmOC facilities and identified catchment population for the facility	NA	CT	NA	NA	NA	NA	NA	1	0	NA	NA	1	1	1	NA	1	NA	CT	NA	NA	NA	1	NA	NA	NA	NA	NA
Identified underserved areas using disaggregated data	NA	1	NA	NA	NA	NA	NA	1	1	NA	NA	1	1	1	NA	1	NA	1	NA	NA	NA	1	NA	NA	NA	NA	NA
Included public and private	NA	1	NA	NA	NA	NA	NA	1	0	NA	NA	1	1	1	NA	1	NA	1	NA	NA	NA	1	NA	NA	NA	NA	NA
**Indicator 3: Proportion of all births in EmOC facilities**																											
Used most recent population size (or projected population if recent population size is older than 5 years)	1	1	NA	1	NA	1	NA	NA	NA	1	NA	NA	1	NA	NA	NA	1	1	1	NA	NA	CT	NA	1	NA	NA	NA
Used disaggregated data to relevant geographical level (national, district, sub-district)	1	1	NA	1	NA	1	NA	NA	NA	1	NA	NA	1	NA	NA	NA	1	1	1	NA	NA	1	NA	1	NA	NA	NA
**Indicator 4: Met need for EmOC**																											
Adhered to operational definition of direct obstetric complications	1	1	NA	NA	NA	1	NA	NA	NA	1	NA	NA	1	NA	NA	NA	1	1	NA	NA	NA	1	NA	NA	NA	CA	1
Defined period for which data on women treated for direct obstetric complications was collected	1	1	NA	NA	NA	1	NA	NA	NA	1	NA	NA	1	NA	NA	NA	1	1	NA	NA	NA	1	NA	NA	NA	CA	1
Used most recent population size (or projected population if recent population size is older than 5 years)	1	1	NA	NA	NA	1	NA	NA	NA	1	NA	NA	1	NA	NA	NA	1	1	NA	NA	NA	CT	NA	NA	NA	1	1
Used disaggregated data to relevant geographical level (national, district, sub-district)	1	1	NA	NA	NA	1	NA	NA	NA	1	NA	NA	1	NA	NA	NA	1	1	NA	NA	NA	1	NA	NA	NA	1	1
**Indicator 5: Caesarean sections as a proportion of all births**																											
Used denominator as the expected number of live births (in the whole catchment area, not just in institutions)	1	1	NA	1	NA	0	0	NA	1	1	NA	1	1	NA	NA	NA	1	1	NA	NA	NA	CT	NA	1	NA	NA	1
Used disaggregated data to relevant geographical level (national, district, sub-district)	1	1	NA	1	NA	1	1	NA	1	1	NA	1	1	NA	NA	NA	1	1	NA	NA	NA	0	NA	1	NA	NA	1
**Indicator 6: Direct obstetric case fatality rate**																											
Used as numerator data of women who developed direct obstetric complications after admission, and die before discharge	1	1	NA	0	NA	1	NA	NA	NA	1	NA	NA	1	NA	NA	NA	1	1	NA	NA	NA	1	NA	1	NA	1	1
Used as denominator number of women who were treated in the same facility and over the same period as numerator	1	1	NA	0	NA	1	NA	NA	NA	1	NA	NA	1	NA	NA	NA	1	1	NA	NA	NA	1	NA	1	NA	1	1
Calculated cause-specific fatality rates for each of the major causes of maternal death	1	1	NA	0	NA	0	NA	NA	NA	0	NA	NA	0	NA	NA	NA	1	0	NA	NA	NA	0	NA	0	NA	1	1
**Indicator 7: Intrapartum and very early neonatal death rate**																											
Used fresh stillbirths (intrapartum and very early neonatal deaths within the first 24 hours) as numerator	1	0	NA	NA	NA	0	NA	NA	NA	NA	NA	NA	1	NA	NA	NA	NA	NA	NA	NA	NA	NA	NA	NA	NA	0	NA
Denominator used was all women who gave birth in the facility during the same period	1	1	NA	NA	NA	1	NA	NA	NA	NA	NA	NA	1	NA	NA	NA	NA	NA	NA	NA	NA	NA	NA	NA	NA	0	NA
Newborns under 2.5 kg were excluded from the numerator and the denominator	CT	0	NA	NA	NA	0	NA	NA	NA	NA	NA	NA	CT	NA	NA	NA	NA	NA	NA	NA	NA	NA	NA	NA	NA	0	NA
**Indicator 8: Proportion of deaths due to indirect causes in EmOC facilities**																											
Used data on 'previous existing disease or disease that developed during pregnancy and which was not due to direct obstetric causes, but which was aggravated by the physiologic effects of pregnancy	1	1	NA	NA	NA	NA	NA	NA	NA	NA	NA	NA	NA	NA	NA	NA	NA	NA	NA	NA	NA	NA	NA	NA	NA	NA	NA
Used as denominator all maternal deaths in the same facilities during the same period	1	1	NA	NA	NA	NA	NA	NA	NA	NA	NA	NA	NA	NA	NA	NA	NA	NA	NA	NA	NA	NA	NA	NA	NA	NA	NA
Used disaggregated data to relevant geographical level (national, district, sub-district)	1	1	NA	NA	NA	NA	NA	NA	NA	NA	NA	NA	NA	NA	NA	NA	NA	NA	NA	NA	NA	NA	NA	NA	NA	NA	NA
**Combined quality score**	18	20	3	5	0	13	3	5	6	13	1	8	18	6	3	3	13	15	5	2	3	11	1	9	1	7	11
**Maximum obtainable score**	20	23	3	10	3	17	5	6	8	14	3	8	20	6	3	3	14	17	5	3	3	17	3	10	3	13	12
**Overall quality percentage score**	90%	87%	100%	50%	0%	76%	60%	83%	75%	93%	33%	100%	90%	100%	100%	100%	93%	88%	100%	67%	100%	65%	33%	90%	33%	54%	92%

***Legend**: 1(Criterion achieved); 0 (Criterion not achieved); CT (Could not tell); NA (Not applicable because indicator was not assessed)*

Classification breakdown

High quality: ≥75%

Medium quality: 50% - 74%

Low quality: <50%
