# Supplementary material for: Assessing emergency obstetric care provision in low- and middle-income countries: a systematic review of the application of global guidelines
Source: Glob Health Action. 2016 Aug 5;9:10.3402/gha.v9.31880. doi: 10.3402/gha.v9.31880 (PMC4976306; doi:10.3402/gha.v9.31880)
Supplement: Assessing emergency obstetric care provision in low- and middle-income countries: a systematic review of the application of global guidelines [file GHA-9-31880-s003.pdf]

Assessing Emergency Obstetric Care Provision in Low and Middle Income Countries: A Systematic Review of the Application of Global Guidelines

Data extraction sheet

Aduragbemi Banke-Thomas, Kikelomo Wright, Olatunji Sonoiki, Oluwasola Banke-Thomas, Babatunde Ajayi, Onaedo Ilozumba and Oluwarotimi Akinola

S/No	Author(s)	Year	Country of study	Scale of study	Specific study site	Number of facilities studied	Stated study objecive(s)	Assessment model	Study design	Data sources
1	Abegunde et al. [14]	2015	Nigeria	Sub-national	20 general hospitals and 39 primary healthcare centers providing delivery services in Bauchi state.	59	To report the availability, utilization, and quality of emergency obstetric care services in Bauchi State, Nigeria.	UN EmOC assessment tool	Cross-sectional facility-based survey	Records of 20 general hospitals and 39 primary healthcare centers providing delivery services.
2	Admasu, Haile-Mariam & Bailey [15]	2011	Ethiopia	National	795 facilities of 806 licensed hospitals and health centers in Ethiopia.	795	To report on the availability and quality of emergency obstetric and newborn care (EmONC) in Ethiopia.	UN EmOC assessment tool	Cross-sectional facility-based survey	Pre-developed standadrized questionnaires were administered in facilities.
3	Alam et al. [16]	2015	Bangladesh	National	BEmOC), 114 private not-for-profit facilities, and 1696 private for profit facilities offering obstetric care services were identified across the country.	2387	To assess the coverage of emergency obstetric care (EmOC) and the availability of obstetric services in Bangladesh	UN EmOC assessment tool	Mixed methods	National health facility assessment
4	Ali et al. [32]	2008	Pakistan	Sub-national	All public health facilities providing EmOC were included (n = 120)	120	To examine if public health care centres in Pakistan's Punjab province comply with minimum recommendations for basic and comprehensive services.	UN EmOC assessment tool	Cross-sectional facility-based survey	Facility data
5	Ameh et al. [37]	2009	Iraq	Sub-national	19 major hospitals in 8 out of the 18 Governorates.	19	To assess the availability of, and challenges to the provision of emergency obstetric care in Iraq	UN EmOC assessment tool	Mixed methods	Self-administered questionnaires, an in-depth interview and a focus group discussion
6	Ameh et al. [17]	2012	6 developing countries: Kenya, Malawi, Sierra Leone, Nigeria, Bangladesh and India	Multi-country with selected districts in each country	378 health facilities spread across Kenya, Malawi, Sierra Leone, Nigeria, Bangladesh and India.	378	To examine the availability, utilization and quality of EmOC in hospitals and health centres providing maternal and newborn care at both BEmOC and CEmOC level and include estimates of population coverage.	UN EmOC assessment tool	Cross-sectional survey	Facility based survey plus Demographic and Health Survey, District Medical Officers in post and the District Health Management Information System.
7	Anwar et al. [33]	2009	Khulna and Sylhet divisions of Bangladesh	Sub-national	First 24 facilities selected from all study districts. Then 41 public sector EmOC facilities in second survey.	14	To explore the quality of care in the public-sector obstetric care facilities in Khulna and Sylhet divisions of the country and to understand the constraints encountered and possible solutions in implementing the CEmOC programme in rural areas.	UN EmOC assessment tool	Mixed methods	Facility based survey plus in-depth interviews
8	Bosomprah et al. [18]	2016	Ghana	National	All 1159 facilities (both public and private) that recorded at least five deliveries per month in 2009 based on the district health management information system.	1159	To provide clear policy directions for gaps in the provision of signal function services and sub-regions requiring priority attention using data from the 2010 Ghana EmOC survey.	UN EmOC assessment tool	Cross-sectional facility-based survey	2010 Ghana EmONC survey data
9	Compaoré et al. [19]	2014	Burkina Faso	National	All district and regional hospitals.	52 (43 district and 9 regional hospitals).	To assess the readiness of district and regional hospitals in Burkina Faso to provide two key CEmOC functions.	UN EmOC assessment tool	Mixed methods	Epidemiologist visiting all district and regional hospitals. Interviews with hospital managers and registers were sources of information
10	Douangphachanh et al. [20]	2010	Laos	Sub-national	All district (30) and provincial hospitals (3) in Borikhamxay, Khammouane, and Savannakhet provinces in Laos.	33	To determine the availability and use of EmOC in provincial and district hospitals in Borikhamxay, Khammouane, and Savannakhet provinces.	UN EmOC assessment tool	Mixed methods	Hospital records reflecting 12 months of facility data and in-depth interviews
11	Duysburgh et al. [38]	2013	Burkina Faso, Ghana and Tanzania	Multi-country with selected districts in each country.	The study took place in PHC facilities in Burkina Faso, Ghana and Tanzania.	6	To measure overall quality of routine antenatal and childbirth care and to identify areas for improvement	UN EmOC assessment tool and Quality of care assessment framework (interpersonal and technical performance and continuity of care) and satisfaction survey	Mixed methods	(i) health facility surveys, (ii) direct observation studies, (iii) satisfaction surveys (exit interviews) and (iv) document reviews of patients records and maternal and child health registers at the health facilities and districts.
12	Echoka et al. [21]	2013	Kenya	Sub-national	40 health facilities offering delivery services in Malindi District, Kenya.	40	To assess the actual existence and functionality of EmOC services at district leve	UN EmOC assessment tool	Cross-sectional facility-based survey	Standard tool was used to interview the in-charge of maternity unit
13	Fakih et al. [22]	2016	Tanzania	Sub-national	224 health facilities in Zanzibar. Sixty four percent of these facilities were government owned; 34 % were privately owned, while 2 % were parastatal.	224	To assess the availability, accessibility and quality of emergency obstetric care services and essential resources available for maternal and child health services in Zanzibar.	UN EmOC assessment tool	Cross-sectional facility-based survey	Key informant interviews, observations, and data extraction
14	Gabrysch et al. [23]	2011	Zambia	National	1131 facilities offering delivery services: 21 second- and third-level hospitals; 69 first-level hospitals; 117 urban health centers; 873 rural health centers; and 50 health posts.	1370	To assess the availability and coverage of emergency obstetric care (EmOC) services in Zambia.	UN EmOC assessment tool	Mixed methods: Secondary data and primary geographical data collection	Zambian Health Facility Census, Zambian Census of Population and Housing and actual facility mapping.
15	Hanson et al. [24]	2013	Tanzania	Sub-national	159 facilities in five districts in rural Southern Tanzania.	159	To describe routine care of- fered during childbirth at dispensaries, health centres and hospitals in rural Southern Tanzania	Safe Motherhood Needs Assessment and the UN EmOC assessment tool	Cross-sectional facility-based survey	Data was collected in March 2009 by trained interviewers.
16	Hirose et al. [25]	2015	Afghanistan	Facility	Herat Regional Hospital	1	To understand the travel delays of women who were in a life- threatening condition at admission to a large maternity hospital in Afghanistan.		Mixed methods: Interviews and primary geographical data collection	Empirical travel times were collected during a cross-sectional survey of women admitted to the maternity ward of Herat Regional Hospital. Face-to-face interviews were conducted mostly before discharge from women and their husbands.
17	Kim et al. [26]	2012	Afghanistan	National	78 first-line referral facilities located in secure areas of Afghanistan.	78	To assess the availability and utilization of emergency obstetric and neonatal care facilities in Afghanistan	UN EmOC assessment tool	Cross-sectional facility-based survey	Modified Averting Maternal Death and Disability Program Needs Assessment Toolkit. Service statistics were abstracted from the National Health Management Information System.
18	Kongnyuy et al. [27]	2009	Malawi	Sub-national	Three districts in the Central Region of Malawi. Survey of all the 73 health facilities (13 hospitals and 60 health centres)	73	To establish a baseline for the availability, utilisation and quality of maternal and neonatal health care services... in three districts in the Central Region of Malawi	UN EmOC assessment tool	Cross-sectional facility-based survey	The Health Facility Rapid Assessment Tool was adapted from the Rapid Assessment used by the Population Council in Kenya.
19	Mezie-Okoye et al. [28]	2012	Nigeria	Facility	12 functional public health facilities out of the existing 19 in Gokana Local Government Area of Rivers State in South-South Nigeria	12	To assess the status of the availability and performance of EmOC in 12 functional public health facilities out of the existing 19 in Gokana Local Government Area of Rivers State in South-South Nigeria.	UN EmOC assessment tool	Cross-sectional facility-based survey	Key Informant Interviews of heads of the health facilities using a semi-structured questionnaire/checklist.
20	Nesbitt et al. [34]	2013	Ghana	Sub-national	All 86 health facilities in seven districts in Brong Ahafo region.	86	To evaluate quality of routine and emergency intrapartum and postnatal care using a health facility assessment	Quality tool that assessed four dimensions: 1) routine delivery care, including labour and immediate postnatal care, 2) emergency obstetric care (EmOC), 3) emergency newborn care (EmNC), and 4) non-medical quality.	Cross-sectional facility-based survey	Health Facility Assessment survey
21	Owens et al. [29]	2014	Zambia	Sub-national	90 health centers and 10 hospitals n Southern Province, Zambia	100	To evaluate the capacity of health facilities in Southern Province, Zambia, to perform routine obstetric care and EmOC	UN EmOC assessment tool	Mixed methods	An expanded set of signal functions for routine care and EmONC was used to assess the facilities' capacity to provide obstetric and neonatal care. Interviews were completed with 172 health workers.
22	Oyerinde et al. [35]	2011	Sierra Leone	National	All public, private, mission, and non-governmental organization hospitals providing maternal and child health services.	145	To determine the availability, utilization, and quality of EmOC services in all regions of Sierra Leone.	UN EmOC assessment tool	Cross-sectional facility-based survey	Questionnaires and checklists developed by the Averting Maternal Death and Disability program at Columbia University, New York, USA, and UN Partners were adapted for local use.
23	Pattinson et al. [39]	2015	South-Africa	Sub-national	All of 53 community health centres and 63 district, 13 regional and 4 tertiary hospitals were visited.	133	To assess the functionality of healthcare facilities with respect to providing the signal functions of basic and comprehensive emergency obstetric care in 12 districts.	UN EmOC assessment tool		
24	Saidu et al. [30]	2013	Nigeria	Sub-national	258 facilities in six of the 16 Local Government Areas (LGAs) of Kwara State Nigeria.	258	To evaluate the levels of emergency obstetrics care signal functions in health facilities in a developing setting with high maternal morbidity and mortality indices and to determine if there are differences between public and private health facilities in terms of availability of these signal functions.	UN EmOC assessment tool	Cross-sectional facility-based survey	State Ministry of Health and National Population Commission office in Kwara State of Nigeria, and interviewer- administered, facility-assessment questionnaire
25	Ueno et al. [40]	2014	Tanzania	Facility	8 health facilities in Moshi Urban District in northern Tanzania.	8	To describe cadres of health care providers who are considered skilled birth attendants in Tanzania, the EmOC signal functions they perform and challenges associated with performance of EmOC signal functions	UN EmOC assessment tool	Cross-sectional study	Questionnaire and health facility assessment forms were used to collect information from 158 participants and health facilities.
26	Utz et al. [36]	2015	Pakistan	Sub-national	32 health facilities in Attock, Gujranwala, Rahim Yar Khan and Khanewal districts of Pakistan's Punjab province.	32	To assess the availability and quality of Emergency Obstetric and Newborn Care in four districts of Punjab.	UN EmOC assessment tool	Cross-sectional facility-based survey	A pre-formatted questionnaire (Rapid assessment tool) to assess availability of signal functions of Emergency Obstetric and Newborn Care, including staffing and equipment, number of births and women with complications, maternal case fatality rate and stillbirth rate.
27	Wilunda et al. [31]	2015	Uganda	Sub-national	All health facilities in Napak and Moroto districts.	13	To establish the availability of maternal and neonatal healthcare services at different levels of health units; to assess their utilisation; and to determine the quality of services provided.	UN EmOC assessment tool	Cross-sectional facility-based survey	Clinical records and registers, interviewing staff and women attending antenatal and postnatal clinics, and by observation.

S/No	Availability of EmOC services			Geographical distribution of EMOC facilities		
	Collected	Results	Signal functions captured	Collected	How	Results
1	Yes	0.52 per 500,000 (Overall). Only 6 (10.2%) of the 59 sampled facilities met the UN requirements for EmOC centers.	Administration of parenteral oxytocics was performed at all facilities. Parenteral antibiotics were provided at 54 (91.5%) facilities. Only 7 BEmOCs (17.9%) provided assisted vaginal delivery, 8 (20.5%) neonatal resuscitation, 9 (23.1%) removal of retained products, and 17 (43.6%) anticonvulsants.	No		
2	Yes	0.6 EmOC facilities per 500 000 population.	Percentage of hospitals and health centers performing the 9 signal functions captured. Reasons given for not providing the signal functions: lack of supplies, equipment, or drugs; no patient presented with an indication for the function; lack of training; and other human resources issues.	Yes	Global information system technology to map the location of those health facilities.	Facilities were concentrated in the center of the country, leaving peripheral areas underserved.
3	Yes	public, 0.4 private not for profit, and 6.1 private fp) per 500,000 individuals. In the public sector, there were 1.0 CEmOC facilities per 500 000 population; varying from 0.9 to 1.4 across the seven administrative divisions. For	In all facility types, performance of assisted vaginal delivery (range 12.2%–48.4%) and use of parenteral anticonvulsants to treat pre-eclampsia/eclampsia (range 48.6%–80.8%) were low. The main reason for non-availability of EmOC services was a lack of specialist/trained providers.	No		
4	Yes	No district in Punjab met the minimum standards laid down by the UN for providing EmOC services.		No		
5	Yes	26.3% (5/19) of hospitals had been able to provide all the 8 signal functions of comprehensive emergency obstetric care in the previous 3 months.	(18/19) were able to provide parenteral oxytocics and perform manual removal of retained placenta, magnesium sulphate for eclampsia was available in 47.4% (9/19) of hospitals, 42.1% (8/19) provided assisted vaginal delivery, 26.5% (5/19) provided blood transfusion and 89.5% (17/19) offered caesarean.	No		
6	Yes	Fewer than 1 in 4 facilities aiming to provide CEmOC were able to offer the nine required signal functions of CEmOC (23.1%) and only 2.3% of health facilities expected to provide BEmOC met the requirement.	The two signal functions least likely to be provided included assisted delivery (17.5%) and manual vacuum aspiration (42.3%). Population indicators were assessed for 31 districts (total population = 15.7 million).	No		
7	Yes	EmOC facilities only satisfied this criterion in the Khulna region in both 2005 (1.36) and 2006/2007 (1.07).				
8	Yes	1.6(CEmOC)<br>0.3/500,000 (BEmOC)<br>153 EmOC facilities short	89 provided all the necessary BEmOC and CEmOC signal functions 3 months prior to the 2010 survey. Only 21% of facility-based births were in fully functioning EmOC facilities.	Yes	Thematic maps were used to provide insight into inequities in service provision.	Subnational analyses based on estimated total pregnancies in each district revealed a pattern of inequity in service provision across the country.
9	Yes	0.58 per 100,000	Two thirds of regional and 20.9% of district hospitals had blood banks. In the other regional hospitals and in most district hospitals it was possible to perform a blood transfusion by calling on volunteers or family members. In six district hospitals, no blood transfusion was possible.	Yes	Not described.	Map of geo-referenced facilities shown
10	Yes	1.73 per 500,000 (CEmOC)<br>3.12 per 500,000 (BEmOC)	32 (97.0%) were providing parental antibiotics and oxytocin; 18 (54.5%) parental sedatives; 31 (93.9%) manual removal of placenta; 25 (75.8%) removal of retained product of placenta; and 16 (48.5%) provided assisted vaginal delivery.	No		
11	Yes*	Not reported	The score for availability of BEmOC signal functions ranged from 0.63 to 0.71 (expressed on a 0–1 scale). Although scores are relatively high, none of the study facilities performed assisted vaginal delivery (in the past 3 months), meaning none of the facilities provided BEmOC services. The absence in all facilities of a vacuum extractor or a forceps supports this finding.	No		
12	Yes	6.2/500,000 (BEmOC 2.5/500 000 CEmOC 3.7/500 000). However, using the strict WHO definition, none of the facilities met the EmOC requirements, since assisted delivery, by vacuum or forceps was not provided in any facility.	Yes with reasons provided.	Yes	This was achieved through mapping of facilities (by collecting geographical coordinates) to identify gaps in geographical distribution of services and acknowledge added barriers such as distance to facilities.	
13	Yes	There are 20 % fewer EmOC facilities in Zanzibar than required. Yet, the number of CEmOC facilities surpassed the required minimum (133 %). Overall, minimum recommended level of EmOC services in Zanzibar has been met by 87 %.	Yes, showed proportion of health facilities in which each signal function was performed during the past 3 months. Also provided detailed caesarean review.	Yes	No details provided.	Basic EmOC facilities were mainly available in North Pemba and South Pemba regions, as well as West Urban regions. CEmOC facilities were mainly concentrated in Urban West (Unguja); North Pemba and South Pemba regions.
14	Yes	1.34 (CEmOC)<br>4.28 (Overall)<br>Few Zambian health facilities provided all basic EmOC signal functions and had qualified health professionals available on a 24-hour basis. Of the 1131 Zambian delivery facilities, 135 (12%) were classified as providing EmOC.	Nearly all of the 90 hospitals had the capacity to provide the EmOC signal functions, although only 65 (72%) offered all 8 functions simultaneously. Fewer hospitals fulfilled the additional staffing criteria; therefore, only 53 hospitals (59%) were classified as offering at least CEmOC-1 and 30 (33%) as offering full CEmOC.	Yes	proportion of the population within 15 km of services—to conform to the UN benchmark of 3 hours of travel time, assuming a walking speed of 5 km per hour. We mapped health facilities and ward areas in the geographic information system platform ArcGIS 9.2 and created circles of 15-km radius around each delivery facility and around EmOC facilities to calculate the proportion of total area covered.	EmOC facilities and health professionals were unevenly distributed between provinces. 86% of the Zambian population was within 15 km of a facility and 48% lived within 15 km of an EmOC facility.
15	Yes	delivery care was offered in all six hos- pitals and 13 health centres.	Only 28% of first-line facilities (dispensaries and health centres) reported offering active management in the third stage of labour (AMTSL). No first-line facility had provided all signal functions for emergency obstetric complications in the previous six months.	No		
16	No			Yes		Amongst 402 women, 82 (20%) had no delay. The median modelled travel time, reported travel time, and delay were 1.0 hour [Q1-Q3: 0.6, 2.2], 3.6 hours [Q1-Q3: 1.0, 12.0], and 2.0 hours [Q1-Q3: 0.1, 9.2], respectively.
17	Yes*	Not reported	Forty-two percent of peripheral facilities did not perform all 9 signal functions required of comprehensive EmONC facilities.	No*		Map of geo-referenced facilities shown, but authors deemed inappropriate due to incomplete representation due to security reasons.
18	Yes	1.6 comprehensive emergency obstetric care (CEmOC) facilities per 500,000 population and 0.8 basic emergency obstetric care (BEmOC) facilities per 125,000 population.	Only 3.3% (2/60) of health centres could do vacuum extractions, 3.3% (2/60) could perform manual vacuum aspiration for retained products of conception, and 35.0% (21/60) could perform manual removal of placenta. Injectable anticonvulsants, oxytocics and antibiotics were provided by 83.3%, 95.0% and 96.7% of health centres, respectively.	Yes	The geographical distribution of these facilities was determined by using district health maps with facilities plotted in them, and by visiting these facilities.	There was no equitable distribution as some rural areas are not covered. Most of the CEmOC were located in the central area of Lilongwe District and three were actually in or near the capital city.
19	Yes	No facility qualified as Basic EmOC, while one had Comprehensive EmOC status.	Signal functions that required supply of medical consumables were performed by more facilities than services that required special training, equipment and maintenance. Only two facilities (16.67%) had the minimum requirement of ≥4 midwives for 24-hour EmOC service; while only 2.2% of expected births occurred at the facilities.	No		
20	Yes*		Parenteral antibiotic 37 (58%), parenteral oxytocin 58 (91%), parenteral anticonvulsant 59 (92%), manual removal of placenta 52 (81%), manual removal of retained products of conception 22 (34%), instrumental delivery 19 (30%), neonatal resuscitaton 51 (80%), blood transfusion 10 (16%), cesarean 9 (14%)	No		
21	Yes*		Comprehensive EmONC was available in only six of 10 hospitals; the remaining four hospitals did not perform all basic EmONC signal functions. None of the 90 health centers performed the basic set of EmONC signal functions.	No		
22	Yes	1.2 facilities per 500 000	The signal functions most commonly not performed were assisted vaginal delivery and manual vacuum aspiration.	Yes	No details provided.	Eastern Province and Southern Province had the lowest coverage. There was an abundance of CEmOC facilities in Western Area District (where the capital city, Freetown, is located).
23	Yes*		Most Community Health Centers could not give parenteral antibiotics (68%), perform manual removal of the placenta (58%), do an assisted delivery (98%) or perform manual vacuum aspiration (96%). Seventeen per cent of community health centres could not bag-and-mask ventilate a neonate. 48% of the District Hospitals could perform all nine CEmOC signal functions.	No		
24	Yes	13.2/500,000 (CEmOC) and 3.3/500,000 (BEmOC)<br>7 facilities (2.5%) met the UN guidelines for BEmOC facilities, and 28 facilities (10.9%) met the standard for a CEmOC facility. Seven (2.6%) of the 76 private health facilities met the UN guidelines as a BEmOC and 24 (31.6%) as a CEmOC. None of the 182 public facilities met the criteria for BEmOCs; however, 4 (2.2%) met the standard for CEmOCs.		No		
25	Yes*		Only two out of the eight facilities provided all of the required EmOC signal functions. While Assistant Medical Officers are expected to perform all the signal functions, only 38% and 13% had performed vacuum extraction or caesarean sections respectively. Very few registered and enrolled nurse-midwives had performed removal of retained products (22 %) or assisted vaginal delivery (24 and 11 %).	No		
26	Yes	0.9 (CEmOC)<br>0.7 (BEmOC)<br>1.6 (Overall)	All required signal functions were available at 4 (22%) CEmOC facilities, and 3 (21%) BEmOC facilities.	No		
27	Yes	3.7 per 500,000 population (CEmOC)<br>0.0 per 500,000 population (BEmOC)	Assisted vaginal delivery and removal of retained products were the most frequently missing signal functions.	No		

S/No	Proportion of all births in EmOC facilities		Met need for EmOC		Caesarean sections as a proportion of all births		Direct obstetric case fatality rate		Intrapartum and very early neonatal death rate		Proportion of deaths due to indirect causes in EmOC facilities		Number of indicators collected
	Collected	Results	Collected	Results	Collected	Results	Collected	Results	Collected	Results	Collected	Results	
1	Yes	Overall, 10 517 (4.4%) of annual births took place in EmOC facilities.	Yes	3.9%. The proportion whose needs were met varied by zone.	Yes	0.2% of the 239 930 expected live births.	Yes	45 (3.2%) of 1416 women with major direct obstetric complications treated at EmOC facilities died.	Yes	Overall, 24 intrapartum and very early neonatal deaths per 1000 deliveries were recorded in the EmOC facilities.	Yes	13.50%	7
2	Yes	3% in EmOC facilities (range, 0%–40% in the regions).	Yes	Nationally, 3% of these were treated in an EmOC facility.	Yes	National average was 0.6% and only 2 regions had rates that fell within UN recommended range (Harari at 9.9% and Addis Ababa at 7.1%).	Yes	direct obstetric complications was 2%. Highest cause-specific case fatality rates: 6.4% (ruptured uterus).	No*	most registers did not differentiate between fresh and macerated stillbirths; and was unable to select for infants who weighed 2500 g or	Yes	Nationally, 21%. Indirect deaths for the regions, which ranged from 7% in Addis Ababa to 48% in Benishangul-Gumuz.	7
3	No		No		No		No		No		No		1
4	Yes	4.70%	No		Yes	0.40%		The case fatality rate was hard to accurately calculate due to poor record keeping and data quality.	No		No		3
5	No		No		No		No		No		No		1
6	Yes	Ranged from 9.9% to 47.5% in 31 districts across five countries.	Yes	Ranged from 6.5% to 35.0%.	Yes	The population based caesarean rate was estimated to be 2%. District level population-based CS rates are low in all countries.	Yes	Facility based case fatality rates are above 1% in all districts surveyed ranging from 2.0% Bangladesh to 9.3% in northern Nigeria.	No*	Stillbirth rates at facility level ranged from 1.9% in Malawi to 6.8% in Sierra Leone.	No		5
7	No		No		Yes*	On average, seven caesarean sections were conducted per month from targeted facilities providing CEmOC in the high-performing areas while the rate was one per month in a low-performing rural comprehensive EOC facility.	No		No		No		2
8	No		No		No		No		No		No		2
9	No		No		Yes	The proportions of caesarean performed in regional versus district hospitals were 19.7% and 10.3% respectively.	No		No		No		2*
10	Yes	11.2% overall. Borikhamxay - 15.5%, Khammouane - 14.7% and Savannakhet - 8.6%	Yes	14.5% overall. 21.0% Khammouane, 18.0% Borikhamxay, and 11.0% Savannakhet.	Yes	The cesarean section rate was only 0.9%. 1.5% in Khuammouane, 0.9% in Borikhamxay, and 0.7% in Savannakhet, respectively.	Yes	Average case fatality rate of three provinces was 0.9%.	No		No		6
11	No		No		No		No		No		No		1
12	No		No		Yes	3.7% in 2008 and 4.5% in 2009. The rates were lower in the rural than in urban areas (2.1% vs. 6.8%; p < 0.001) in 2008 and (2.7% vs. 7.7%; p < 0.001) in 2009.	No		No		No		3
13	Yes	47 % (25,560/54,057) of all births occurred in EmONC health facilities	Yes	33.1 % (2519/7354). Unguja zone had a higher met need of 33.7 % (1396/4137). Urban District had the highest met need at 55.2 % (1315/2383)	Yes	4.4% of expected births in Zanzibar were cesarean deliveries. Unguja zone had a slightly higher caesarean delivery rate. The highest caesarean rate was in Urban District at 23.9%.	Yes	Overall direct obstetric case fatality rate was 2.5% for all health facilities surveyed.	Yes	2% (n = 553) overall, with inter-district variation. The highest being in South and Urban districts with 5.8% (n = 46) and 2.4% (n = 377) respectively.	No		7
14	No		No		No		No		No		No		2
15	No		No		No		No		No		No		1
16	No		No		No		No		No		No		1
17	Yes	17%	Yes	20%	Yes	1%	Yes	0.80%	No		Yes	0.20%	5
18	Yes	About 23% of deliveries were conducted in emergency obstetric care (EmOC) facilities.	Yes	The met need for emergency obstetric complications was 20.7%.	Yes	2.7 (870/31,637) - Population based	Yes	The case fatality rate for emergency obstetric complications treated in health facilities was 2.0%.	No		No		6
19	Yes	2.20%	No		No		No		No		No		2*
20	No		No		No		No		No				1
21	No		No		No		No		No		No		1
22	Yes	Only 10% of expected births in Sierra Leone occurred in a health facility of any type, with 2% occurring in an EmOC facility.	Yes	7% nationally. Only Western Province had a met need above 10%.	Yes	Less than 1% nationally.	Yes	The national CFR was 7% but, because of poor record keeping, this statistic is unreliable.	No		No		6
23	No		No		No		No		No		No		1*
24	Yes	13.60%	No		Yes	2.30%	Yes	The resulting obstetric case fatality rate was 8.7% within the state and 9.3% for EOC facilities	No		No		5
25	No		No		No		No		No		No		
26	No		Yes	17.80%			Yes	Overall case fatality rate for all districts of 1.75% with disease-specific case fatality rates also estimated.	Yes*	The total number of stillbirths in the preceding 3 months was 257 with an estimated stillbirth rate of 30 per 1000 births.	No		4
27	No		Yes	9.90%	Yes	1.70%	Yes	3%	No		No		4

Acronymns	
AMDD	Averting Maternal Death and Disability
AMTSL	Active Management in the Third Stage of Labour
BEmOC	Basic Emergency Obstetric Care
CEmOC	Basic Emergency Obstetric Care
EmOC	Emergency Obstetric Care
EmONC	Emergency Obstetric and Newborn Care
GIS	Geographic Information Systems
UN	United Nations

References

[14] Admasu K, Haile-Mariam A, Bailey P. Indicators for availability, utilization, and quality of emergency obstetric care in Ethiopia, 2008. Int J Gynaecol Obstet 2011;115:101–5.
[15] Abegunde D, Kabo IA, Sambisa W, Akomolafe T, Orobaton N, Abdulkarim M, et al. Availability, utilization, and quality of emergency obstetric care services in Bauchi State, Nigeria. Int J Gynaecol Obstet 2015;128:251–5.
[16] Alam B, Mridha MK, Biswas TK, Roy L, Rahman M, Chowdhury ME. Coverage of emergency obstetric care and availability of services in public and private health facilities in Bangladesh. Int J Gynaecol Obstet 2015;131:63–9.
[17] Ameh C, Msuya S, Hofman J, Raven J, Mathai M, van den Broek N. Status of emergency obstetric care in six developing countries five years before the MDG targets for maternal and newborn health. PLoS One 2012;7:e49938.
[18] Bosomprah S, Tatem AJ, Dotse-Gborgbortsi W, Aboagye P, Matthews Z. Spatial distribution of emergency obstetric and newborn care services in Ghana: Using the evidence to plan interventions. Int J Gynaecol Obstet 2016;132:130–4.
[19] Compaoré GD, Sombié I, Ganaba R, Hounton S, Meda N, Brouwere V De, et al. Readiness of district and regional hospitals in Burkina Faso to provide caesarean section and blood transfusion services: a cross-sectional study. BMC Pregnancy Childbirth 2014;14:158.
[20] Douangphachanh X, Ali M, Outavong P, Alongkon P, Sing M, Chushi K. Availability and use of emergency obstetric care services in public hospitals in Laos PDR: a systems analysis. Biosci Trends 2010;4:318–24.
[21] Echoka E, Kombe Y, Dubourg D, Makokha A, Evjen-Olsen B, Mwangi M, et al. Existence and functionality of emergency obstetric care services at district level in Kenya: theoretical coverage versus reality. BMC Health Serv Res 2013;13:113.
[22] Fakih B, Nofly AAS, Ali AO, Mkopi A, Hassan A, Ali AM, et al. The status of maternal and newborn health care services in Zanzibar. BMC Pregnancy Childbirth 2016;16:134.
[23] Gabrysch S, Simushi V, Campbell OMR. Availability and distribution of, and geographic access to emergency obstetric care in Zambia. Int J Gynaecol Obstet 2011;114:174–9.
[24] Hanson C, Ronsmans C, Penfold S, Maokola W, Manzi F, Jaribu J, et al. Health system support for childbirth care in Southern Tanzania: results from a health facility census. BMC Res Notes 2013;6:435.
[25] Hirose A, Borchert M, Cox J, Alkozai AS, Filippi V. Determinants of delays in travelling to an emergency obstetric care facility in Herat, Afghanistan: an analysis of cross-sectional survey data and spatial modelling. BMC Pregnancy Childbirth 2015;15:14.
[26] Kim Y-M, Zainullah P, Mungia J, Tappis H, Bartlett L, Zaka N. Availability and quality of emergency obstetric and neonatal care services in Afghanistan. Int J Gynaecol Obstet 2012;116:192–6.
[27] Kongnyuy EJ, Hofman J, Mlava G, Mhango C, van den Broek N. Availability, utilisation and quality of basic and comprehensive emergency obstetric care services in Malawi. Matern Child Health J 2009;13:687–94.
[28] Mezie-Okoye MM, Adeniji FO, Tobin-West CI, Babatunde S. Status of emergency obstetric care in a local government area in south-south Nigeria. Afr J Reprod Health 2012;16:171–9.
[29] Owens L, Semrau K, Mbewe R, Musokotwane K, Grogan C, Maine D, et al. The state of routine and emergency obstetric and neonatal care in Southern Province, Zambia. Int J Gynaecol Obstet 2015;128:53–7.
[30] Saidu R, August EM, Alio AP, Salihu HM, Saka MJ, Jimoh AAG. An assessment of essential maternal health services in Kwara State, Nigeria. Afr J Reprod Health 2013;17:41–8.
[31] Wilunda C, Oyerinde K, Putoto G, Lochoro P, Dall'Oglio G, Manenti F, et al. Availability, utilisation and quality of maternal and neonatal health care services in Karamoja region, Uganda: a health facility-based survey. Reprod Health 2015;12:30.
[32] Ali M, Ahmed KM, Kuroiwa C. Emergency obstetric care in Punjab, Pakistan: improvement needed. Eur J Contracept Reprod Health Care 2008;13:201–7.
[33] Anwar I, Kalim N, Koblinsky M. Quality of obstetric care in public-sector facilities and constraints to implementing emergency obstetric care services: evidence from high- and low-performing districts of Bangladesh. J Health Popul Nutr 2009;27:139–55.
[34] Nesbitt RC, Lohela TJ, Manu A, Vesel L, Okyere E, Edmond K, et al. Quality along the continuum: a health facility assessment of intrapartum and postnatal care in Ghana. PLoS One 2013;8:e81089.
[35] Oyerinde K, Harding Y, Amara P, Kanu R, Shoo R, Daoh K. The status of maternal and newborn care services in Sierra Leone 8years after ceasefire. Int J Gynecol Obstet 2011;114:168–73.
[36] Utz B, Zafar S, Arshad N, Kana T, Gopalakrishnan S. Status of emergency obstetric care in four districts of Punjab, Pakistan - results of a baseline assessment. J Pak Med Assoc 2015;65:480–5.
[37] Ameh CA, Bishop S, Kongnyuy E, Grady K, Van den Broek N. Challenges to the provision of emergency obstetric care in Iraq. Matern Child Health J 2009;15:4–11.
[38] Duysburgh E, Zhang W-H, Ye M, Williams A, Massawe S, Sié A, et al. Quality of antenatal and childbirth care in selected rural health facilities in Burkina Faso, Ghana and Tanzania: similar finding. Trop Med Int Health 2013;18:534–47.
[39] Pattinson RC, Makin JD, Pillay Y, Broek N van der, Moodley J. Basic and comprehensive emergency obstetric and neonatal care in 12 South African health districts. South African Med J 2015;105:256–60.
[40] Ueno E, Adegoke AA, Masenga G, Fimbo J, Msuya SE. Skilled birth attendants in Tanzania: a descriptive study of cadres and emergency obstetric care signal functions performed. Matern Child Health J 2015;19:155–69.
